# Age-related differences in the clinical features of ocular sarcoidosis

**DOI:** 10.1371/journal.pone.0202585

**Published:** 2018-08-23

**Authors:** Kei Takayama, Kozo Harimoto, Tomohito Sato, Yutaka Sakurai, Manzo Taguchi, Takayuki Kanda, Masaru Takeuchi

**Affiliations:** Department of Ophthalmology, National Defense Medical College, Tokorozawa, Japan; Oregon Health and Science University, UNITED STATES

## Abstract

The distribution of age at diagnosis in ocular sarcoidosis has shifted towards the older age groups in developed countries. In systemic sarcoidosis, age-related differences in the clinical presentation, which reflect the therapeutic strategies, was reported. We retrospectively compared 100 consecutive patients from April 2010 to March 2016 who were initially diagnosed with ocular sarcoidosis by International Workshop on Ocular Sarcoidosis criteria. They were classified into elder (>65 years: 50 patients) and younger (≤65 years: 50 patients) groups by the age at diagnosis of uveitis associated with sarcoidosis. All patients received ophthalmic examination to assess the presence of seven intraocular signs and 4 laboratory parameters. Significantly fewer ocular signs (2.8 ± 1.5 and 3.6 ± 1.5; P = 0.0034) and abnormal laboratory results (1.5 ± 1.2 and 2.0 ± 1.2; P = 0.023) were detected in the elder group than in the younger group; statistical differences were found between the groups regarding the frequencies of mutton-fat keratic precipitates (40% and 64%; P = 0.012), vitreous opacities (60% and 78%; P = 0.0059), bilateral inflammation (64% and 80%; P = 0.012), and bilateral hilar lymphadenopathy between the groups (52% and 78%; P < 0.001). Multiple linear regression analysis showed negative correlations between age and number of detected ocular signs (r = −0.36, P < 0.001) and laboratory results (r = −0.20, P = 0.023). The characteristic ocular signs and abnormal laboratory results had a lower frequency in the elder patients compared with the younger patients. Probable or possible ocular sarcoidosis by the international criteria should increase with increased life expectancy in developed countries.

## Introduction

Sarcoidosis is a chronic inflammatory disease characterised by the formation of non-caseating granulomas in multiple tissues and organs, including the eye [1, 2]. The prevalence of ocular involvement found in different series ranges widely from 13% (Turkish study) [3] to 79% (Japanese study) of patients with systemic sarcoidosis [4], and females (56%) are more likely to develop ocular involvement than males (23%) [5]. The distribution of age at diagnosis has shifted towards older age groups in developed countries such as the United States, European countries and Japan [6]. Therefore, its incidence has decreased among young people, although there has consistently been a second peak in postmenopausal women [7].

The gold standard for diagnosis of sarcoidosis involves a biopsy of the relevant tissue, such as the lung, skin or lymph nodes [8]. However, biopsy procedures are not acceptable for uveitis patients associated with sarcoidosis, and intraocular tissue biopsies are not generally performed because they are invasive and can result in vision loss [9]. In addition, although usefulness of conjunctival and lacrimal gland biopsies for diagnosing ocular sarcoidosis have been reported [10, 11], these are not sensitive for sarcoidosis patients with uveitis. In 2009, the International Workshop on Ocular Sarcoidosis (IWOS) drafted a set of guidelines for characterising uveitis patients suspected of having sarcoidosis. These guidelines designate four diagnostic categories: definite, presumed, probable or possible ocular sarcoidosis [12]. Although diagnosis of definite ocular sarcoidosis also requires biopsy [13, 14], effort has been made to determine which clinical signs and laboratory results are useful for correctly diagnosing sarcoidosis in patients with uveitis.

Age-related differences in the clinical presentation of systemic sarcoidosis have been reported [6]. Extrathoracic lymph node involvement is more common in young patients, whereas extrathoracic involvement of non-lymphatic organs and hypercalcaemia are more frequent in older patients. The age-related differences in the clinical presentation of sarcoidosis may reflect the pathways of the causative antigens and the strengthening of immunoregulatory mechanisms with age [7]. Nevertheless, the age-related differences in the manifestation of symptoms in ocular sarcoidosis have not been reported. The aim of this study was to compare the background and frequency of clinical ocular signs and laboratory investigations as assigned by the IWOS criteria between younger and older patients with ocular sarcoidosis and to determine the age-related characteristics of this pathology.

## Methods

### Patients

Consecutive clinical records of 100 patients in whom ocular sarcoidosis was diagnosed with the initial development of uveitis according to the IWOS criteria at the National Defense Medical College Hospital from April 2010 to July 2016 were reviewed. The National Defense Medical College Hospital Ethics Review Board approved this retrospective analysis of patient data (2853). This study was described to all human subjects, and written informed consent was waived by the ERB due to the retrospective nature of the study. Retirement system is commonly conducted in Japan, and the retirement age is 65 years old in the most of companies. Therefore, patients were classified into elder (>65 years: 50 patients) and younger (≤65 years: 50 patients) groups by the age at diagnosed as uveitis associated with sarcoidosis. Based on the IWOS criteria, the patients were diagnosed with definite, presumed, probable or possible ocular sarcoidosis. Patients who did not meet one of the IWOS categories were excluded from the study. Exclusion criteria were presence of corneal diseases, primary glaucoma, exfoliation syndrome, history of trauma or surgery to the globe, history of other uveitis, other systemic inflammation disease, malignancy, or systemic corticosteroid and/or anti-immunological agent intake. In addition, because biopsy was not performed for all sarcoidosis patients, especially for older patients, definite cases of ocular sarcoidosis were also excluded. Patients who had undergone small-incision cataract surgery more than 1 year ago were included.

### Ocular signs and laboratory examinations

The IWOS clinical intraocular signs and laboratory examinations are listed in Table 1.

**Table 1:** The International Workshop on Ocular Sarcoidosis criteria.

Ocular signs
Keratic precipitates/iris nodules
Trabecular meshwork nodules and/or tent-shaped peripheral anterior synechiae
Vitreous opacities displaying snowballs/strings of pearls
Multiple chorioretinal peripheral lesions
Nodular and/or segmental periphlebitis and/or retinal macroaneurysm
Optic disc nodule/granuloma and/or solitary choroidal nodule
Bilateral inflammation
Laboratory examinations
Negative tuberculin purified protein derivative skin test
Elevated serum angiotensin-converting enzyme and/or serum lysozyme
Bilateral hilar lymphadenopathy
Elevated liver enzyme tests

Ocular signs were counted if they were present in at least one eye. In Laboratory examinations, patients who were suspected of bilateral hilar lymphadenopathy (BHL) on chest X-radiograph (CXR) by radiologists were referred to respiratory physician, and received additional chest computed tomography (CT) scan. CT scan was not performed for patients with and without apparent BHL on CXR.

### Outcomes and statistical analysis

The number of ocular and systemic signs were evaluated, and the chi-squared and Mann–Whitney U tests were used to compare results between the two groups. The Spearman’s rank correlation coefficient was used to analyse correlations between age and other data, and a multiple linear regression analysis was then used to detect the correlations between age and other parameters. P values > 0.05 were considered statistically significant.

## Results

### Characteristics and differences in the ocular and laboratory signs between the two groups

**Fig 1:**
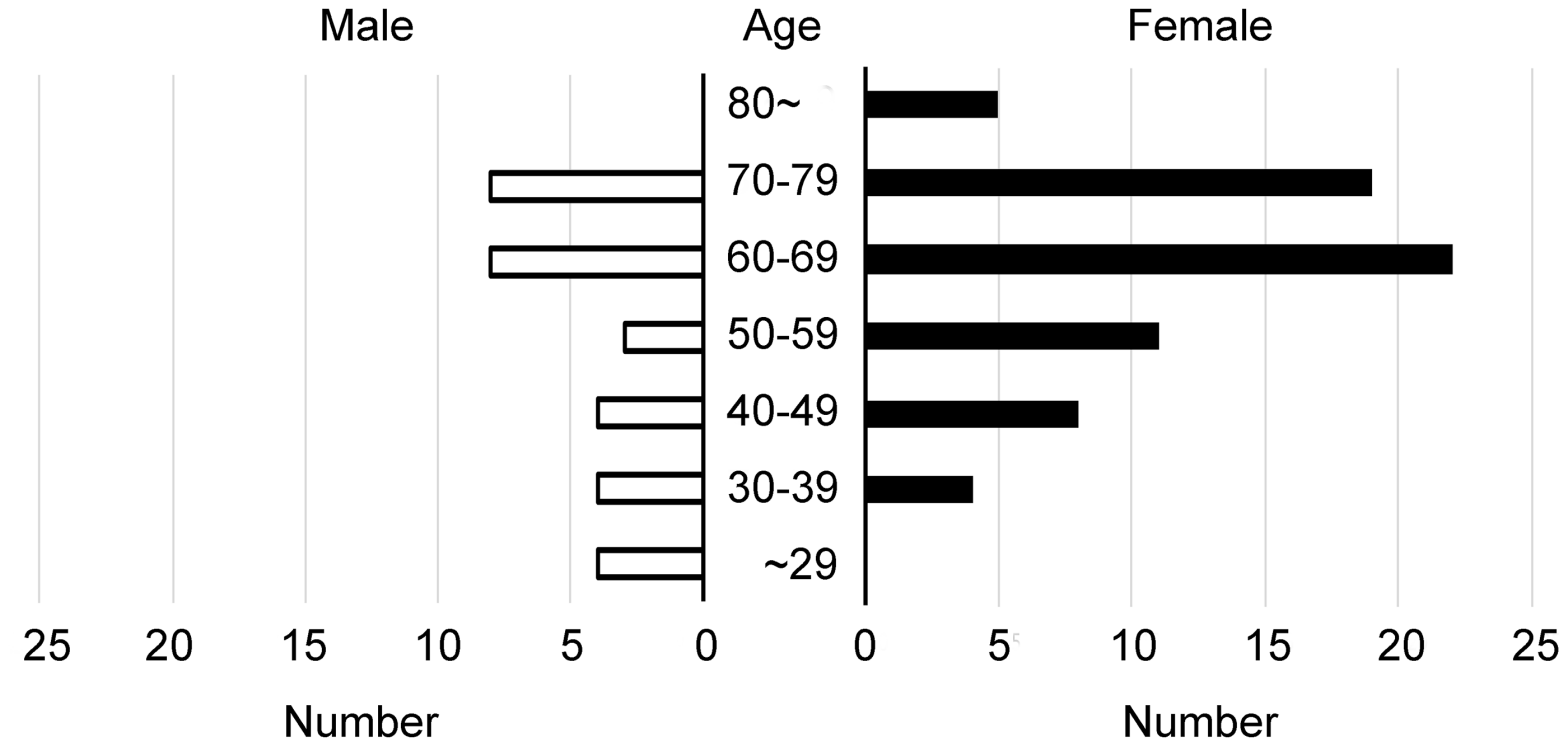
Classification of patients with ocular sarcoidosis by age. The numbers of patients were 4, 8, 12, 14, 30, 27, and 5 in the under 30s, 30s, 40s, 50s, 60s, 70s, and over 80s, respectively.

The numbers of patients classified into each age group are shown in Fig 1.

For both males and females, the most common age group was the 60s group (8 out of 30 males and 22 out of 70 females), followed by the 70s group (8 out of 30 males and 19 out of 70 females). Patient characteristics and sensitivity of the ocular and systemic signs in the elder and younger groups are shown in Table 2.

**Table 2:** Patient characteristics and sensitivity of the clinical signs.

	Elder	Younger	P^#^
Number	50	50	
Male/female	18/32	13/37	
Mean age (years)	48.7 ± 11.6	72.8 ± 5.8	
Diagnosis of ocular sarcoidosis			
Presumed	26	39	
Probable	14	7	
Possible	10	4	
Ocular signs			
Keratic precipitates/iris nodules	40%	64%	0.012
Trabecular meshwork nodules and/or tent-shaped peripheral anterior synechiae	20%	28%	0.19
Vitreous opacities displaying snowballs/strings of pearls	60%	78%	0.0059
Multiple chorioretinal peripheral lesions	32%	36%	0.55
Periphlebitis and/or macroaneurysm	54%	58%	0.57
Optic disc nodule and/or choroidal nodule	10%	12%	0.65
Bilateral inflammation	64%	80%	0.012
Laboratory investigations			
Negative tuberculin purified	88%	80%	0.12
Elevated serum ACE and/or lysozyme	26%	36%	0.13
Bilateral hilar lymphadenopathy	52%	78%	<0.001
Elevated liver enzyme tests	30%	34%	0.54

^#^: analysed by 2 × 2 chi-squared test and Fischer’s exact test

In the elder group, 26 patients, 14 patients and 10 patients were diagnosed as having presumed, probable and possible ocular sarcoidosis, respectively. In the younger group, 39 patients, 7 patients and 4 patients were diagnosed as having presumed, probable and possible ocular sarcoidosis, respectively. For the defined ocular signs, the frequencies of keratic precipitates (KP), vitreous opacities (VO) and bilateral inflammation were significantly higher in the younger group (64%, 78% and 80%, respectively) than in the elder group (40%, 60% and 64%, respectively). For the laboratory examinations, BHL was significantly more common in the younger group (78%) than in the elder group (52%). We then compared the numbers of positive ocular signs and laboratory investigations between the two groups (Table 3).

**Table 3:** Numbers of positive ocular signs and laboratory investigations.

	Elder	Younger	P*
Ocular signs (7 signs)	2.8 ± 1.5	3.6 ± 1.4	0.0034
Laboratory investigations (4 signs)	1.5 ± 1.2	2.0 ± 1.2	0.023
All (11 signs)	4.3 ± 2.2	5.5 ± 2.1	0.0021

*: analysed by the Mann–Whitney U test

Of the seven ocular signs, the mean number of positive ocular signs was 2.8 ± 1.5 in the elder group and 3.6 ± 1.4 in the younger group, and there was a statistical difference between them (P = 0.0034). Additionally, of the four laboratory investigations, the mean number of positive laboratory investigations in the elder group was 1.5 ± 1.2, which was significantly lower than that found in the younger group (2.0 ± 1.2; P = 0.023).

### Correlations between age and distinguishing signs

**Fig 2:**
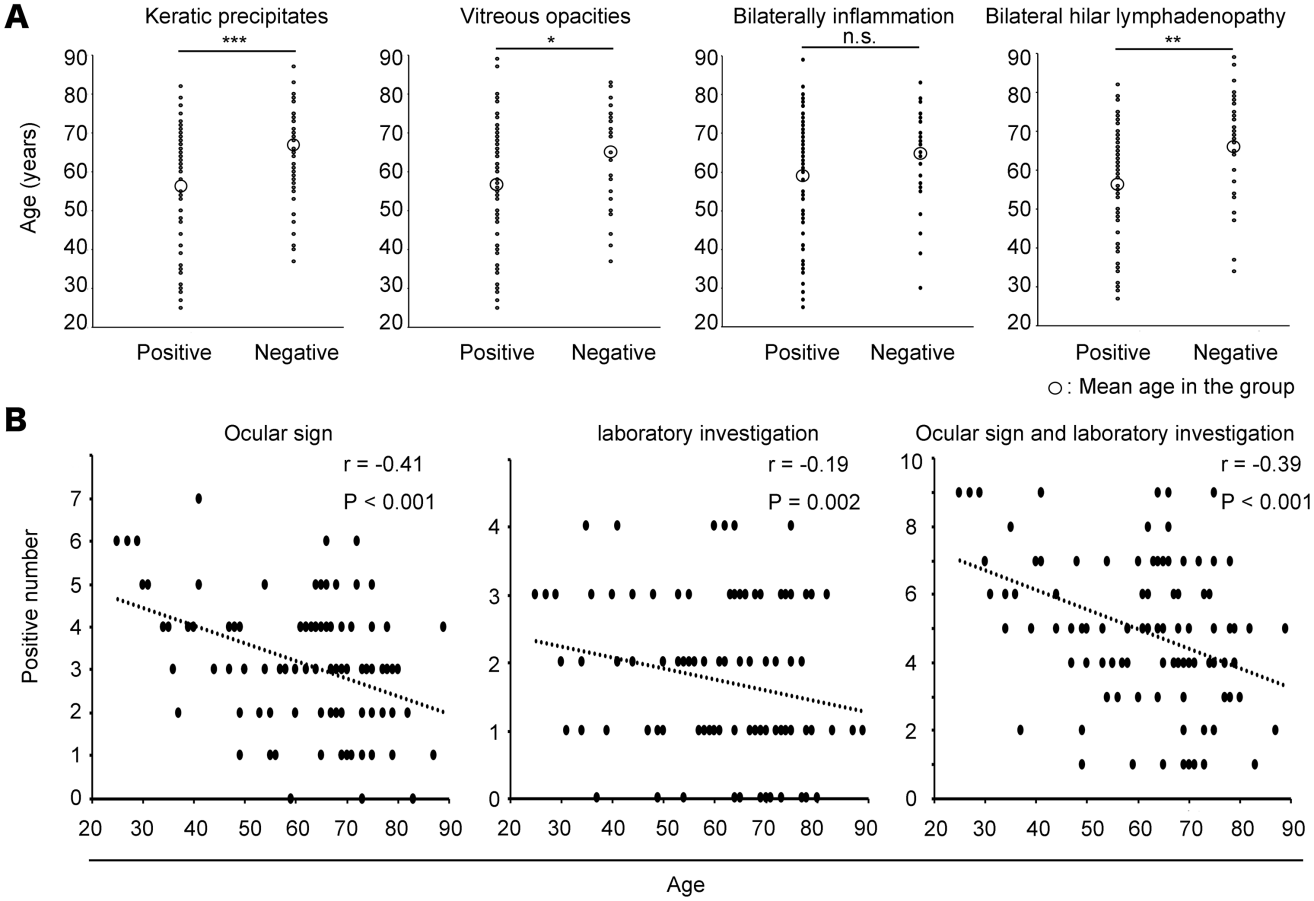
Age differences and correlations between age and ocular signs, laboratory investigation. **(A)** Dot plots of each patient’s age with and without KP, VO, bilateral inflammation or BHL. The mean age of the patients with KP, VO or BHL was significantly lower than that of patients without. **(B)** The age of the patients was negatively correlated with the number of positive ocular signs, positive laboratory investigations and total signs. *: P < 0.05, **: P < 0.01, ***: P < 0.001.

Fig 2 shows dot plots of the age of each patient with and without KP, VO, bilateral inflammation or BHL.

The patients with KP (56.0 ± 15.9 years), VO (58.5 ± 15.7 years) and BHL (57.5 ± 15.4 years) were significantly younger than those without KP (65.9 ± 12.6 years), VO (65.8 ± 12.8 years) and BHL (66.6 ± 13.0 years), although there was no significant difference in the mean age of the patients with and without bilateral inflammation (59.1 ± 15.7 and 65.0 ± 13.0 years, respectively; P = 0.08). Subsequently, the age and the number of positive ocular signs, laboratory investigations or total number of signs were correlated using multiple linear regression analysis. Patient age was negatively correlated with the number of positive ocular signs (r = −0.36, P < 0.001), positive laboratory results (r = −0.20, P = 0.023) and total number of signs (r = −0.36, P < 0.001).

## Discussion

In the present study, we compared the presence of the characteristic ocular signs and laboratory investigations for ocular sarcoidosis between elder and younger patients, and our results indicated that the frequencies of KP, VO and BHL are lower in elderly patients than in younger patients, and that the frequencies decrease on an age-dependent basis.

Over time, the age distribution at diagnosis of sarcoidosis has shifted towards the older age groups in the developed countries [6]. Previous study has reported that most patients in their 20s present BHL, however this is consistently less common among older patients [7]. In the present study, BHL was significantly less in elder patients compared with younger patients, which is consistent with the previous study. As well, KP and VO were less observed in elderly patients, and negative correlations between age and ocular signs or laboratory investigations were demonstrated (Fig 2). The tendency of less characteristic ocular signs among elderly patients would be the same as systemic signs in the previous systemic sarcoidosis report. Age-related changes of immunological mechanism have been indicated including natural killer T (NKT) cells, and immune function decreases in elder people [15, 16]. NKT cells are integral components of immune responses during many chronic diseases including sarcoidosis [17, 18], and NKT cells in peripheral blood correlate with the suppression of sarcoidosis activity [19]. In lung, NKT cells also activate and regulate immunological system in bronchoalveolar lavage fluid [20].

The age distribution at the diagnosis of sarcoidosis is biphasic for women, with a second peak after 45 years of age that has been consistently shown in Japan over the last four decades [7]. In the present study, there was a peak appearing in the 60s age group in both males and females. Regarding females after the 50s age, sarcoidosis onset could potentially be accelerated by insidious ovarian dysfunction associated with menopause. Epidemiological research carried out in the U.S. Black Women’s Health Study suggested that endogenous female hormones could possibly protect against the onset of sarcoidosis [21]. Early clinical observations among women with a previous diagnosis of sarcoidosis have revealed postpartum relapse as well as remission during pregnancy, suggesting that certain female reproductive and hormonal factors may reduce the disease’s activity. Based on experimental findings on enhanced granulomatous reactions after bilateral oophorectomy [22, 23], decreases in the circulating ovarian hormones promote the development of granulomatous diseases, including sarcoidosis.

In older males and females after age 60, the environmental factors in addition to age-related changes might also increase possibility of sarcoidosis development. The U.S.-based ACCESS (A Case Control Etiologic Study of Sarcoidosis) study identified occupational exposure to insecticides, pesticides and mould/mildew to have positive associations with a modest 1.5-fold increased risk of sarcoidosis; agricultural employment and environments with possible exposure to microbial bioaerosols [24]. The Standard Industrial Classification and Standard Occupational Classification tools support positive associations of sarcoidosis risk with occupational exposure related to elementary/secondary schools, building materials/garden supplies/mobile homes/hardware and industrial organic dusts [25]. These studies support the hypothesis that age-related hormone changes and environmental exposure influence sarcoidosis susceptibility.

Tissue biopsy is a useful method, although it is invasive and has some risks [26, 27]. The age distribution has shifted towards the older age groups, and elderly patients may not tolerate biopsy because of their systemic condition. In addition, our results indicated that elderly patients show fewer positive signs of ocular and laboratory investigations. Therefore, diagnosing ocular sarcoidosis has become more difficult.

Some limitations of the present study that should be noted are the small number of patients only from Japanese origin, the single-centre, and retrospective nature of the study. Further prospective study including more patients from other ethnicities as well as evaluations of therapeutic effects could allow additional comparison of differences in ocular sarcoidosis between elderly and younger patients.

## Conclusion

Our results indicated that the characteristic ocular signs and abnormal laboratory results had a lower frequency in elder patients compared with younger patients. Probable or possible ocular sarcoidosis by the international criteria should increase with increased life expectancy in developed countries. Further study to find novel symptom manifestations may be helpful in the diagnosis of ocular sarcoidosis in elderly patients.
